# The possible impact of increased physical intimate partner violence during the COVID-19 pandemic on ocular health

**DOI:** 10.1177/20503121211035263

**Published:** 2021-07-31

**Authors:** Patrice M Hicks, Maureen A Murtaugh, Margaret M DeAngelis

**Affiliations:** 1 Department of Population Health Sciences, University of Utah, Salt Lake City, UT, USA; 2 Department of Ophthalmology and Visual Sciences, University of Utah, Salt Lake City, UT, USA; 3 Department of Ophthalmology, University of Buffalo, Buffalo, NY, USA; 4 VA Western New York Healthcare System, Buffalo, NY, USA

**Keywords:** Epidemiology/public health, ophthalmology, women’s health, COVID-19, pandemic

## Abstract

During the COVID-19 outbreak, sheltering at home has led to an increase in physical intimate partner violence cases. Intimate partner violence–sustained ocular injuries may be higher during the pandemic due to the increase in physical intimate partner violence. Left untreated, intimate partner violence–related ocular or orbital trauma can lead to permanent vision loss. Even with treatment, patients often lose vision from intimate partner violence–related traumatic ocular injuries. Eye care providers and eye care facilities should understand the community services available to intimate partner violence survivors to better care for these patients. Due to the potential lasting economic burden and social strain of this pandemic, eye care providers and facilities should stay vigilant as there may still be a sustained increase in intimate partner violence even after the global COVID-19 pandemic.

## Introduction

COVID-19 was declared a pandemic by the World Health Organization in March 2020 and continues to have significant impacts.^1–3^ The novel coronavirus SARS-CoV-2 causes an acute atypical respiratory disease.^4,5^ Individuals at a greater risk for COVID-19 are older and have underlying medical conditions that are not well-controlled.^6^ Moreover, those who are a part of the black and brown community are more likely to experience health burdens in COVID-19. This increased risk may be due to reduced access to health care services, decreased ability to work from home, higher poverty rates, and living in cities with greater numbers of COVID-19 cases.^7,8^

Around the globe, restrictions and orders have been put into place to enforce social distancing to combat the spread of COVID-19.^9,10^ These restrictions include stay-at-home orders or sheltering in place to decrease the spread of this virus.^11,12^ In addition, many occupations are considered non-essential during the pandemic, and many are not able to work remotely.^13,14^ Though the restrictions put into place help decrease a person’s risk for getting the novel virus and decrease the spread of the virus to others, these restrictions and orders may still negatively impact a person’s health. As more individuals spend time at home due to these restrictions, there has been an increase in physical intimate partner violence (IPV).^15–17^ A IPV incident can cause ocular and orbital injury, leading to permanent damage to an individual’s vision.^18,19^ In this review, we will explore how increased rates of IPV, including physical IPV, during COVID-19 can have lasting effects on ocular health and what could be implemented.

## Intimate partner violence

The Centers for Disease Control and Prevention states that intimate partner violence is sexual violence, physical violence, stalking, or psychological harm done by either a current or former spouse or partner. IPV can occur among heterosexual or same-sex couples and does not require sexual intimacy.^20^ IPV is a severe public health problem that affects millions of Americans and has negative individual and social outcomes.^21–23^ Almost 20% of women report that an intimate partner has caused severe physical violence in their lifetime.^20^ It is estimated that 1.3 million women are victims of physical IPV each year.^24^ Data throughout the years show that IPV increases during pandemics and economic crises.^25–27^ Almost 41% of women IPV survivors experience a form of physical injury related to IPV.^20^ Previous research has found that trauma to the eye can cause glaucoma, cataracts, retinal hemorrhaging, and retinal detachment.^28–31^ However, there is little research focusing on how IPV impacts eye health.^32–34^ Information on mechanisms of orbital and ocular trauma and secondary outcomes such as traumatic glaucoma and ruptured globes remains poorly explored.^32,35^

Racial and ethnic minorities are disproportionally burdened by IPV.^36–38^ Ethnic minority women experience a more significant burden of accessing help for IPV and secondary outcomes than non-Hispanic white women.^39,40^ Minority women who experience IPV are less likely to have access to services, including health care, due to higher rates of poverty, language barriers, fear of deportation, and limited job opportunities.^41–43^ Further research is needed to understand the prevalence of ocular or orbital trauma due to IPV and the potential associated risk factors for ocular or orbital trauma in this underserved population. Moreover, research is needed to develop interventions better to decrease blindness due to IPV ocular or orbital trauma health in underserved populations.^44^

Such as with COVID-19,^45–47^ IPV disproportionally burdens individuals from lower socioeconomic status. Insurance coverage which varies with socioeconomic status and employment status can influence likelihood of COVID diagnosis and treatment.^48,49^ IPV outcomes in terms of both mental and physical health are worse for those with lower-socioeconomic status.^39,50^ The homes may not be a safe space for individuals who experience IPV during the COVID-19 pandemic.^25,51^ The COVID-19 pandemic could initiate IPV in households where it did not occur or worsen during this time due to economic uncertainty, decreased access to community services, and increased stress due to the unknown.^52,53^

## Increase of IPV during COVID-19

Cases of IPV have increased globally during the pandemic (Table 1). IPV is one of the unintentional adverse outcomes of implementing stay-at-home orders to prevent the spread of COVID-19.

**Table 1.** Increase in intimate partner violence (IPV) during global COVID-19 pandemic.

Continent	Increase in IPV
Africa	• 87,000 cases of gender-based violence in South Africa during the first week of stay-at-home orders.^54,55^
Asia	• In China, IPV reports tripled during their stay-at-home orders compared to the previous year.^56^ • India had a 100% increase in gender-based violence complaints in April 2020.^57^
Australia	• 5% increase in IPV calls.^58^ • 75% increase in Google search related to support for IPV.^58^
Europe	• Italian and French government commissioned hotels to provide shelter to those escaping IPV situations.^58^ • 30% increase in IPV cases in France.^9^ • UK had a 25% increase in IPV calls.^58^
North America	• USA overall increase by state ranging from 21% to 35%.^58^ • Canada’s COVID response package included funding to support women’s shelters for those experiencing gender-based violence.^59^ • In Mexico, 911 related domestic violence calls increased by 60%, 3 weeks after social distancing orders.^60^
South America	• 40%–50% increase in IPV cases in Brazil.^9^ • In Peru, the incidence rate of calls for IPV was 9% greater.^61^ • Argentina had a 25% increase in calls.^62^

The factors that influence IPV could be exacerbated during the pandemic. Individuals who rely on their partner financially may find it more difficult during the pandemic to have a stable income or the ability to find employment.^63^ In addition, IPV survivors may feel isolated and without support systems including family and friends^64–66^ which can aid in them successfully leaving their abuser.^67–69^ Women who experience IPV are more likely to experience mental health problems.^70^ It is already stressful for IPV survivors to leave their abusive situation and planning to leave takes a lot of thought and effort because it can be dangerous.^71,72^

It is critical that health care providers, including eye care providers, assess individuals for IPV as they may access less care during this time. IPV is a leading cause of homelessness for women.^73,74^ Those who experience IPV may be more reluctant to stay at an IPV shelter because of the increased COVID-19 spread in community living situations.^75^ Access to health care services, including eye care services, has been reduced during the pandemic.^76,77^ This decreases the chances that an individual who has experienced IPV will come into contact with a health care provider who could provide necessary ocular treatment or additional IPV resources. Group therapy for IPV is effective, but social distancing orders can create a participation barrier.

## Ocular health and domestic violence

Forty-five percent of IPV injury incidents involve the eye.^32^ Orbital fractures are the most common eye injury associated with IPV incidents. In women, 7.6% of orbital floor fractures were due to IPV.^33^ In a study that included both men and women, researchers found that orbital fractures made up 13% of fracture locations in patients with IPV-related fractures.^78^ Additional research is needed to identify the prevalence of orbital fractures due to IPV in men because men tend to underreport their IPV experiences.^79^

Annually 1.6 million people lose their sight due to ocular injury.^80–82^ Although orbital fractures are more common, ruptured globes result in more severe outcomes including severe vision loss and blindness and psychological impacts.^83,84^ More research is also needed to determine how often ocular trauma, glaucoma, traumatic cataracts, retinal hemorrhages, or retinal detachments occur and how often permanent loss of vision or blindness occur.^28–32,85,86^

Vision loss is associated with secondary outcomes including depression, decreased mobility, a more significant economic burden, independence, and decreased life quality.^44^ Eye care for IPV survivors may prevent lifelong secondary outcomes from ocular trauma.^87^ However, poor access to eye care services reduces timely care for orbital fractures and ocular trauma due to an IPV incident.^88,89^ Delayed care also reduces access to social resources for the individuals who have experienced IPV.

## Addressing IPV and ocular health during a pandemic

Health care providers from all fields, including eye care providers, will need to remain vigilant during this pandemic and beyond. Specifically, eye care providers for patients with ocular trauma incidents should screen patients for IPV and be aware of local community resources.^90^ Community centers should have eye care providers to increase likelihood that eye and vision care services are received by those who may not be able to easily access care due to travel barriers, insurance coverage, cost, lack of services, or lack of culturally competent care.^91,92^

Screening for IPV and providing intervention should be within the scope of practices of eye care providers and eye care facilities.^91,92^ Understanding the available resources is essential to the ability of the eye care providers and eye care facilities to address IPV.^91,93^ Eye care providers should refer those who have experienced IPV to counseling, hotlines, shelters, rape crisis centers, or health care facility social workers and counselors to improve their well-being.^94,95^ Ensuring that these connections are local will better serve the individual who has experienced IPV.^96,97^

Before the pandemic, IPV was already underreported and underrecognized by health care professionals, so it is essential with the growing numbers that each provider and facility address this.^98–100^ Eye care providers should also train their medical team on IPV.^101,102^ Training on IPV impacts on health outcomes, specifically related to ocular eye health, is also needed.^103^ Addressing IPV during the COVID-19 pandemic and beyond should be a team-based effort to ensure IPV survivors have the needed tools and health care.^104^

## Limitations

Prior research studies relevant to this review are limited but highlight the need for more research within this field. Also, many people who experience IPV may be hesitant to report, so that IPV cases may be underreported. Furthermore, IPV may be challenging to assess as, in many cases, this is written in the free-text area of the electronic health records and more difficult to quantify. This review highlights the need to understand IPV and incorporate appropriate screening and treatment during a global pandemic and after.

## Conclusion

IPV cases have increased globally since the COVID-19 pandemic. Eye care providers and eye care facilities should be vigilant that there can be a lasting increase in IPV cases due to the lasting economic and social strains that may occur even after the COVID-19 pandemic. Eye care providers can aid in assisting the growing number of individuals who have experienced IPV in multiple ways. Eye care providers should assess patients for physical IPV-related injuries that may have lasting effects on their vision and eye care needs even if treated. Eye care providers and eye and health care facilities should provide those with IPV-related injuries with community support tools.

## References

[1] Spinelli A Pellino G. COVID-19 pandemic: perspectives on an unfolding crisis. Br J Surg 2020; 107(7): 785–787. 32191340 10.1002/bjs.11627 PMC7228411

[2] World Health Organization. Coronavirus disease (COVID—19)—events as they happen, https://www.who.int/emergencies/diseases/novel-coronavirus-2019/events-as-they-happen (accessed 27 July 2020).

[3] World Health Organization. WHO Director-General’s opening remarks at the media briefing on COVID-19 - 11 March 2020, https://www.who.int/dg/speeches/detail/who-director-general-s-opening-remarks-at-the-media-briefing-on-covid-19—11-march-2020 (accessed 27 July 2020).

[4] Yuki K Fujiogi M Koutsogiannaki S. COVID-19 pathophysiology: a review. Clin Immunol 2020; 215: 108427. 32325252 10.1016/j.clim.2020.108427 PMC7169933

[5] Lai CC Shih TP Ko WC , et al. Severe acute respiratory syndrome coronavirus 2 (SARS-CoV-2) and coronavirus disease-2019 (COVID-19): the epidemic and the challenges. Int J Antimicrob Agents 2020; 55(3): 105924. 32081636 10.1016/j.ijantimicag.2020.105924 PMC7127800

[6] Centers for Disease Control and Prevention. People who are at increased risk for severe illness, https://www.cdc.gov/coronavirus/2019-ncov/need-extra-precautions/people-at-higher-risk.html (accessed 27 July 2020).

[7] Laurencin CT McClinton A. The COVID-19 pandemic: a call to action to identify and address racial and ethnic disparities. J Racial Ethn Health Disparities 2020; 7(3): 398–402. 32306369 10.1007/s40615-020-00756-0 PMC7166096

[8] Gould E Shierholz H. Not everybody can work from home: Black and Hispanic workers are much less likely to be able to telework, https://www.epi.org/blog/black-and-hispanic-workers-are-much-less-likely-to-be-able-to-work-from-home/ (accessed 27 July 2020).

[9] Campbell AM. An increasing risk of family violence during the Covid-19 pandemic: strengthening community collaborations to save lives. Foren Sci Int Rep 2020; 2: 100089. 10.1016/j.fsir.2020.100089 PMC7152912 38620174

[10] Sen-Crowe B McKenney M Elkbuli A. Social distancing during the COVID-19 pandemic: staying home save lives. Am J Emerg Med 2020; 38(7): 1519–1520. 32305155 10.1016/j.ajem.2020.03.063 PMC7194642

[11] Mervosh S Lu D Swales V. See which states and cities have told residents to stay at home, https://www.nytimes.com/interactive/2020/us/coronavirus-stay-at-home-order.html (accessed 27 July 2020).

[12] Tull MT Edmonds KA Scamaldo KM , et al. Psychological outcomes associated with stay-at-home orders and the perceived impact of COVID-19 on daily life. Psychiatry Res 2020; 289: 113098. 32434092 10.1016/j.psychres.2020.113098 PMC7252159

[13] Baron S McPhaul K Phillips S , et al. Protecting home health care workers: a challenge to pandemic influenza preparedness planning. Am J Public Health 2009; 99(Suppl. 2): S301–S307. 19461108 10.2105/AJPH.2008.157339 PMC4504355

[14] Gostin LO Wiley LF. Governmental public health powers during the COVID-19 pandemic: stay-at-home orders, business closures, and travel restrictions. JAMA 2020; 323(21): 2137–2138. 32239184 10.1001/jama.2020.5460

[15] Moreira DN da Costa MP. The impact of the Covid-19 pandemic in the precipitation of intimate partner violence. Int J Law Psychiatry 2020; 71: 101606. 32768122 10.1016/j.ijlp.2020.101606 PMC7318988

[16] Roesch E Amin A Gupta J , et al. Violence against women during covid-19 pandemic restrictions. BMJ 2020; 369: m1712. 32381644 10.1136/bmj.m1712 PMC7202944

[17] Gosangi B Park H Thomas R , et al. Exacerbation of physical intimate partner violence during COVID-19 pandemic. Radiology 2021; 298(1): E38–E45. 32787700 10.1148/radiol.2020202866 PMC7427119

[18] Beck SR Freitag SL Singer N. Ocular injuries in battered women. Ophthalmology 1996; 103(1): 148–151. 8628545 10.1016/s0161-6420(96)30748-3

[19] Goldberg SH McRill CM Bruno CR , et al. Orbital fractures due to domestic violence: an epidemiologic study. Orbit 2000; 19(3): 143–154. 12045943 10.1076/orbi.19.3.143.2659

[20] Centers for Disease Control and Prevention. Preventing intimate partner violence, https://www.cdc.gov/violenceprevention/intimatepartnerviolence/fastfact.html (accessed 24 February 2020).

[21] Spivak HR Jenkins L VanAudenhove K , et al. CDC Grand Rounds: a public health approach to prevention of intimate partner violence. MMWR Morb Mortal Wkly Rep 2014; 63(2): 38–41. 24430100 PMC4584651

[22] Dutton MA James L Langhorne A , et al. Coordinated public health initiatives to address violence against women and adolescents. J Womens Health 2015; 24(1): 80–85. 10.1089/jwh.2014.4884 PMC4302966 25549182

[23] Weil A. Intimate partner violence: epidemiology and health consequences. Walthman, MA: UpToDate, 2016.

[24] The National Domestic Violence Hotline. Seeing beyond abuse, https://www.thehotline.org/2010/11/11/seeing-beyond-abuse/ (accessed 24 February 2020).

[25] Valera E. When lockdown is not actually safer: intimate partner violence during COVID-19, https://www.health.harvard.edu/blog/when-lockdown-is-not-actually-safer-intimate-partner-violence-during-covid-19-2020070720529 (accessed 27 July 2020).

[26] Enarson E. Violence against women in disasters: a study of domestic violence programs in the United States and Canada. Violence Against Women 1999; 5(7): 742–768.

[27] Schneider D Harknett K McLanahan S. Intimate partner violence in the great recession. Demography 2016; 53(2): 471–505. 27003136 10.1007/s13524-016-0462-1 PMC4860387

[28] Sihota R Kumar S Gupta V , et al. Early predictors of traumatic glaucoma after closed globe injury: trabecular pigmentation, widened angle recess, and higher baseline intraocular pressure. Arch Ophthalmol 2008; 126(7): 921–926. 18625937 10.1001/archopht.126.7.921

[29] Shah MA Shah SM Shah SB , et al. Morphology of traumatic cataract: does it play a role in final visual outcome? BMJ Open 2011; 1(1): e000060. 10.1136/bmjopen-2011-000060 PMC3191399 22021742

[30] Johnston PB. Traumatic retinal detachment. Br J Ophthalmol 1991; 75(1): 18–21. 1991080 10.1136/bjo.75.1.18 PMC504099

[31] Levin AV. Retinal hemorrhage in abusive head trauma. Pediatrics 2010; 126(5): 961–970. 20921069 10.1542/peds.2010-1220

[32] Cohen AR Renner LM Shriver EM. Intimate partner violence in ophthalmology: a global call to action. Curr Opin Ophthalmol 2017; 28(5): 534–538. 28549018 10.1097/ICU.0000000000000397

[33] Berrios DC Grady D. Domestic violence risk factors and outcomes. West J Med 1991; 155(2): 133–135. 1926841 PMC1002942

[34] Kaur R Garg S. Addressing domestic violence against women: an unfinished agenda. Indian J Community Med 2008; 33(2): 73–76. 19967027 10.4103/0970-0218.40871 PMC2784629

[35] Coker AL Smith PH Fadden MK. Intimate partner violence and disabilities among women attending family practice clinics. J Womens Health 2005; 14(9): 829–838. 10.1089/jwh.2005.14.829 16313210

[36] Baral SD Rucinski KB Rwema JOT , et al. The relationship between the global burden of influenza from 2017-2019 and COVID-19, https://www.medrxiv.org/content/10.1101/2020.06.18.20134346v1 10.2196/24696 PMC7927952 33522974

[37] Centers for Disease Control and Prevention. Health equity considerations and racial and ethnic minority groups, https://www.cdc.gov/coronavirus/2019-ncov/need-extra-precautions/racial-ethnic-minorities.html (accessed 27 July 2020).

[38] Townsend MJ Kyle TK Stanford FC. Outcomes of COVID-19: disparities in obesity and by ethnicity/race. Int J Obes 2020; 44(9): 1807–1809. 10.1038/s41366-020-0635-2 PMC7347050 32647359

[39] Stockman JK Hayashi H Campbell JC. Intimate partner violence and its health impact on ethnic minority women. J Womens Health 2015; 24(1): 62–79. 10.1089/jwh.2014.4879 PMC4302952 25551432

[40] Williams JK Wyatt GE Myers HF , et al. Patterns in relationship violence among African American women: future research and implications for intervention. J Aggress Maltreat Trauma 2008; 16(3): 296–310. 24482570 10.1080/10926770801925726 PMC3904364

[41] Women’s Institute for Leadership Development for Human Rights. The treatment of women of color under U.S. law: violence, http://www.wildforhumanrights.org/publications/treatmentwomen/p4.html

[42] Townes C. How women of color are disproportionately impacted by domestic violence, https://thinkprogress.org/how-women-of-color-are-disproportionately-impacted-by-domestic-violence-6674e93a50c5/ (accessed 24 February 2020).

[43] Jones F. Ray Rice: Black women struggle more with domestic abuse, https://time.com/3313343/ray-rice-black-women-domestic-violence/ (accessed 24 February 2020).

[44] National Academies of Sciences, Engineering, and Medicine; Health and Medicine Division; Board on Population Health and Public Health Practice; Committee on Public Health Approaches to Reduce Vision Impairment and Promote Eye Health; Welp A Woodbury RB McCoy MA , et al. Making eye health a population health imperative: vision for tomorrow. Washington, DC: National Academies Press, 2016. 27656731

[45] Patel JA Nielsen FBH Badiani AA , et al. Poverty, inequality and COVID-19: the forgotten vulnerable. Public Health 2020; 183: 110–111. 32502699 10.1016/j.puhe.2020.05.006 PMC7221360

[46] Cardoso MR Cousens SN de Góes Siqueira LF , et al. Crowding: risk factor or protective factor for lower respiratory disease in young children? BMC Public Health 2004; 4: 19. 15176983 10.1186/1471-2458-4-19 PMC434510

[47] Algren MH Ekholm O Nielsen L , et al. Associations between perceived stress, socioeconomic status, and health-risk behaviour in deprived neighbourhoods in Denmark: a cross-sectional study. BMC Public Health 2018; 18(1): 250. 29439681 10.1186/s12889-018-5170-x PMC5812195

[48] King JS. Covid-19 and the need for health care reform. New Engl J Med 2020; 382: 26. 10.1056/NEJMp2000821 32302074

[49] Rollston RL Galea S. The coronavirus does discriminate: how social conditions are shaping the COVID-19 pandemic, http://info.primarycare.hms.harvard.edu/blog/social-conditions-shape-covid (accessed 27 July 2020).

[50] Aizer A. Poverty, violence and health: the impact of domestic violence during pregnancy on newborn health. J Hum Resour 2011; 46(3): 518–538. 24839303 10.1353/jhr.2011.0024 PMC4019993

[51] Bradbury-Jones C Isham L. The pandemic paradox: the consequences of COVID-19 on domestic violence. J Clin Nurs 2020; 29(13–14): 2047–2049. 32281158 10.1111/jocn.15296 PMC7262164

[52] Van Gelder N Peterman A Potts A , et al. COVID-19: reducing the risk of infection might increase the risk of intimate partner violence. EClinicalMedicine 2020; 21: 100348. 32292900 10.1016/j.eclinm.2020.100348 PMC7151425

[53] Abramson A. How COVID-19 may increase domestic violence and child abuse, https://www.apa.org/topics/covid-19/domestic-violence-child-abuse (accessed 27 July 2020).

[54] Joska JA Andersen L Rabie S , et al. COVID-19: increased risk to the mental health and safety of women living with HIV in South Africa. AIDS Behav 2020; 24: 2751–2753. 32347405 10.1007/s10461-020-02897-z PMC7188491

[55] Chothia A. Lockdown: 87 000 cases of gender-based violence reported, https://www.thesouthafrican.com/news/gender-based-violence-reported-during-lockdown-cele/ (accessed 31 August 2020).

[56] Fraser E. Impact of COVID-19 pandemic on violence against women and girls. London: UK Department for International Development, 2020.

[57] Vora M Malathesh BC Das S , et al. COVID-19 and domestic violence against women. Asian J Psychiatr 2020; 53: 102227. 32574942 10.1016/j.ajp.2020.102227 PMC7295494

[58] Usher K Bhullar N Durkin J , et al. Family violence and COVID-19: increased vulnerability and reduced options for support. Int J Ment Health Nurs 2020; 29(4): 549–552. 32314526 10.1111/inm.12735 PMC7264607

[59] United Nations. The impact of COVID-19 on women, https://www.un.org/sites/un2.un.org/files/policy_brief_on_covid_impact_on_women_9_april_2020.pdf (accessed 31 August 2020).

[60] Fernández Nieto B . Domestic violence in Mexico in times of COVID-19, https://datapopalliance.org/domestic-violence-in-mexico-in-times-of-covid-19/ (accessed 31 August 2020).

[61] Agüero JM. COVID-19 and the rise of intimate partner violence, 2020, https://aguero.econ.uconn.edu/wp-content/uploads/sites/1904/2020/11/wd2020.pdf

[62] Boserup B McKenney M Elkbuli A. Alarming trends in US domestic violence during the COVID-19 pandemic. Am J Emerg Med 2020; 38: 2753–2755. 32402499 10.1016/j.ajem.2020.04.077 PMC7195322

[63] Salamone N. Domestic violence and financial dependency, https://www.forbes.com/2010/09/02/women-money-domestic-violence-forbes-woman-net-worth-personal-finance.html (accessed 27 July 2020).

[64] Banerjee D Rai M. Social isolation in Covid-19: the impact of loneliness. Int J Soc Psychiat 2020; 66: 525–527. 32349580 10.1177/0020764020922269 PMC7405628

[65] Centers for Disease Control and Prevention. Mental health and coping during COVID-19, https://www.cdc.gov/coronavirus/2019-ncov/daily-life-coping/managing-stress-anxiety.html (accessed 27 July 2020).

[66] Ducharme J. COVID-19 is making America’s loneliness epidemic even worse, https://time.com/5833681/loneliness-covid-19/ (accessed 27 July 2020).

[67] Machisa MT Christofides N Jewkes R. Social support factors associated with psychological resilience among women survivors of intimate partner violence in Gauteng, South Africa. Glob Health Action 2018; 11(suppl. 3): 1491114. 30270774 10.1080/16549716.2018.1491114 PMC6179050

[68] Dutton MA Goodman LA. Coercion in intimate partner violence: toward a new conceptualization. Sex Roles 2005; 52(11–12): 743–756.

[69] Centers for Disease Control and Prevention. Risk and protective factors for perpetration, https://www.cdc.gov/violenceprevention/intimatepartnerviolence/riskprotectivefactors.html (accessed 27 July 2020).

[70] Temple JR Weston R Marshall LL. Long term mental health effects of partner violence patterns and relationship termination on low-income and ethnically diverse community women. Partner Abuse 2010; 1(4): 379–398. 21755020 10.1891/1946-6560.1.4.379 PMC3133447

[71] Campbell JC Webster D Koziol-McLain J , et al. Risk factors for femicide in abusive relationships: results from a multisite case control study. Am J Public Health 2003; 93(7): 1089–1097. 12835191 10.2105/ajph.93.7.1089 PMC1447915

[72] Mayo Clinic. Recognize the signs of domestic violence against women, https://www.mayoclinic.org/healthy-lifestyle/adult-health/in-depth/domestic-violence/art-20048397 (accessed 27 July 2020).

[73] Thurston WE Roy A Clow B , et al. Pathways into and out of homelessness: domestic violence and housing security for immigrant women. J Immig Refug Stud 2013; 11(3): 278–298.

[74] Administration for Children and Families. Domestic violence and homelessness: statistics, 2016, https://www.acf.hhs.gov/fysb/resource/dv-homelessness-stats-2016 (accessed 27 July 2020).

[75] Maxmen A. Coronavirus is spreading under the radar in US homeless shelters, https://www.nature.com/articles/d41586-020-01389-3 (accessed 27 July 2020). 10.1038/d41586-020-01389-3 32393874

[76] Centers for Disease Control and Prevention. Healthcare facilities: managing operations during the COVID-19 pandemic, https://www.cdc.gov/coronavirus/2019-ncov/hcp/guidance-hcf.html?permalink=63CDAEE3D21BD3386CECE970773F32603322B1877DEEDF7BD3FC710459FA4269 (accessed 27 July 2020).

[77] Mehrotra A Chernew M Linetsky D , et al. The impact of the COVID-19 pandemic on outpatient visits: a rebound emerges, https://www.commonwealthfund.org/publications/2020/apr/impact-covid-19-outpatient-visits

[78] Porter A Montgomery CO Montgomery BE , et al. Intimate partner violence-related fractures in the United States: an 8 year review. J Fam Violence 2019; 34(7): 601–609. 32595265 10.1007/s10896-018-0007-z PMC7318917

[79] Carmo R Grams A Magalhães T. Men as victims of intimate partner violence. J Forensic Leg Med 2011; 18(8): 355–359. 22018167 10.1016/j.jflm.2011.07.006

[80] Cao H Li L Zhang M. Epidemiology of patients hospitalized for ocular trauma in the Chaoshan region of China, 2001–2010. PLoS ONE 2012; 7(10): e48377. 23118997 10.1371/journal.pone.0048377 PMC3485239

[81] Négrel AD Thylefors B. The global impact of eye injuries. Ophthalmic Epidemiol 1998; 5(3): 143–169. 9805347 10.1076/opep.5.3.143.8364

[82] Whitcher JP Srinivasan M Upadhyay MP. Corneal blindness: a global perspective. Bull World Health Organ 2001; 79(3): 214–221. 11285665 PMC2566379

[83] Coulibaly TA Béogo R Traoré I , et al. Inter personal violence-related facial injuries: a 10-year survey. J Oral Med Oral Surg 2018; 24(1): 2–5.

[84] Grob SR Talcott KE Stryjewski T , et al. Intimate partner violence: an important etiology to identify in patients with open globe injuries. Investig Ophthalmol Vis Sci 2017; 58(8): 5516.

[85] Bojikian KD Stein AL Slabaugh MA , et al. Incidence and risk factors for traumatic intraocular pressure elevation and traumatic glaucoma after open-globe injury. Eye 2015; 29(12): 1579–1584. 26381097 10.1038/eye.2015.173 PMC5129804

[86] Osman EA. Glaucoma after open globe injury. Saudi J Ophthalmol 2015; 29(3): 222–224. 26155083 10.1016/j.sjopt.2014.10.006 PMC4487842

[87] Chowdhury S. Injuries in marginal workers and social trauma in female: important cause of the paradigm shift in eye injury over a decade. Indian J Occup Environ Med 2015; 19(1): 36–43. 26023270 10.4103/0019-5278.157006 PMC4446937

[88] Ross JS Bradley EH Busch SH. Use of health care services by lower-income and higher-income uninsured adults. JAMA 2006; 295: 2027–2036. 16670411 10.1001/jama.295.17.2027

[89] Lee DJ Lam BL Arora S , et al. Reported eye care utilization and health insurance status among US adults. Arch Ophthalmol 2009; 127(3): 303–310. 19273794 10.1001/archophthalmol.2008.567 PMC11025701

[90] Miller E McCaw B Humphreys BL , et al. Integrating intimate partner violence assessment and intervention into healthcare in the United States: a systems approach. J Womens Health 2015; 24(1): 92–99. 10.1089/jwh.2014.4870 PMC4302956 25606823

[91] HealthyPeople.gov. Access to health services, 2020, https://www.healthypeople.gov/2020/topics-objectives/topic/Access-to-Health-Services (accessed 27 July 2020).

[92] Hamberger LK Rhodes K Brown J. Screening and intervention for intimate partner violence in healthcare settings: creating sustainable system-level programs. J Womens Health 2015; 24(1): 86–91. 10.1089/jwh.2014.4861 PMC4302953 25412012

[93] Usta J Taleb R. Addressing domestic violence in primary care: what the physician needs to know. Libyan J Med 2014; 9: 23527. 10.3402/ljm.v9.23527 PMC3957738 24647277

[94] Sullivan CM. Understanding how domestic violence support services promote survivor well-being: a conceptual model. J Fam Violence 2018; 33(2): 123–131. 29367804 10.1007/s10896-017-9931-6 PMC5760592

[95] Bennett L Riger S Schewe P , et al. Effectiveness of hotline, advocacy, counseling, and shelter services for victims of domestic violence: a statewide evaluation. J Interpers Violence 2004; 19(7): 815–829. 15186538 10.1177/0886260504265687

[96] Shorey RC Tirone V Stuart GL. Coordinated community response components for victims of intimate partner violence: a review of the literature. Aggress Violent Behav 2014; 19(4): 363–371. 25089115 10.1016/j.avb.2014.06.001 PMC4113829

[97] Temple KM. Domestic and intimate partner violence: some do’s and don’ts for health providers, https://www.ruralhealthinfo.org/rural-monitor/domestic-violence/ (accessed 27 July 2020).

[98] Dicola D Spaar E. Intimate partner violence. Am Fam Phys 2016; 94(8): 646–651. 27929227

[99] Wygant CRC Bruera E Hui D. Intimate partner violence in an outpatient palliative care setting. J Pain Symptom Manage 2014; 47(4): 806–813. 23948161 10.1016/j.jpainsymman.2013.05.018 PMC3844013

[100] Fife R Schrager S. Family violence: what health care providers need to know. Burlington, MA: Jones & Bartlett Publishers, 2012, p. 99.

[101] Bair-Merritt MH Lewis-O’Connor A Goel S , et al. Primary care–based interventions for intimate partner violence: a systematic review. Am J Prev Med 2014; 46(2): 188–194. 24439354 10.1016/j.amepre.2013.10.001

[102] Fawole OI Balogun BO Adejimi AA , et al. Training medical students: victim’s perceptions of selectively screening women for intimate partner violence in health care settings. BMC Med Educ 2019; 19(1): 196. 31185978 10.1186/s12909-019-1627-6 PMC6558861

[103] Clark TJ Shriver EM. Intimate partner violence (IPV) awareness: ASK, ASSESS, REFER, http://EyeRounds.org/tutorials/IPV.htm (accessed 23 March 2021).

[104] Decker MR Frattaroli S McCaw B , et al. Transforming the healthcare response to intimate partner violence and taking best practices to scale. J Womens Health 2012; 21(12): 1222–1229. 10.1089/jwh.2012.4058 PMC3654819 23210490

